# Sensory Characteristics of Various Concentrations of Phenolic Compounds Potentially Associated with Smoked Aroma in Foods

**DOI:** 10.3390/molecules23040780

**Published:** 2018-03-28

**Authors:** Hongwei Wang, Edgar Chambers

**Affiliations:** 1 College of Food Science, Southwest University, Chongqing 400715, China; wanghw_1978@swu.edu.cn; 2 Center for Sensory Analysis and Consumer Behavior, Kansas State University, Manhattan, KS 66502, USA

**Keywords:** phenolic, odor, smoked, smoky, sensory, aroma

## Abstract

This research describes the sensory odor characteristics of 19 phenolic compounds (11 phenol derivatives, six guaiacol derivatives, and two syringol derivatives) that have been associated with smoked aroma in previous literature. Seven concentrations varying from 1 to 100,000 ppm of each chemical were examined. A highly trained descriptive panel used a recently published lexicon for smoky aroma and flavor and found that smoked aroma compounds have many different attributes that make up smokiness. Musty/dusty, musty/earthy, pungent, acid, smoky, woody, burnt, ashy, cedar, creosote or petroleum-like collectively imparted smoked aroma. Most of the phenolic compounds were described as having smoky characteristics at low concentrations, generally at 1 and 10 ppm, except 3,4-dimethylphenol at 5000 ppm. 2,6-Dimethylphenol was not associated with smoky characteristics. This research is the first to evaluate a set of phenolic compounds for their sensory characteristics using a professionally developed set of sensory attributes.

## 1. Introduction

The smoking process has a considerable influence on the sensory characteristics of smoked products that are demanded by consumers [1,2]. Many studies have been carried out to characterize the volatile compound compositions of smoked products, wood smokes and smoke flavorings [3,4,5,6,7,8,9,10,11]. More recently, studies focused on phenolic compounds have shown their development and deposition depends on the smoking processes [12,13] and they are regarded as the main source of flavor and aroma in smoke and smoked products. Kostyra and Baryłko-Pikielna [10] suggested that phenol, *p*-cresol and *o*-cresol seem to play the most important role in the smoke-curing profile while contributions of guaiacol and 4-methylguaiacol are possible. Cardinal et al. [14] investigated the relationships between sensory properties and phenolic compounds by multiple linear models. Varlet et al. [15] suggested that phenolic compounds, such as cresol or guaiacol, seem to be responsible for smoked odor by comparing the odor-active volatile compounds of fresh and smoked salmon. Stojković et al. [16] indicated that phenols contributed to the smoke flavor of sheep ham. Smoke taint of wines was thought to be closely related to the volatile phenol composition and glycoconjugated phenols [17,18,19,20,21,22,23]. Fudge et al. [24] used reverse osmosis and solid phase adsorption to reduce the concentration of volatile phenols and ameliorate smoke taint in wine.

Smoked flavor is not a single smoky characteristic, but rather has several different manifestations, such as wood or wood fire [1,11,25], rubber [1,26,27], and others. Jaffe et al. [28] used a highly trained and skilled descriptive sensory panel to identify, define and reference 14 sensory attributes related to the flavor that is imparted to products during the smoking process. The lexicon included terms such as smoky, ashy, woody, musty/dusty, musty/earthy, burnt, acrid, pungent, petroleum-like, creosote/tar, cedar, bitter, metallic and sour.

Many studies have described the smoky aroma characteristics of volatile phenolic compounds by gas chromatography-olfactometry methods and gas chromatography-mass spectrometry. There are some different descriptive items of a chemical, for example, 2-methoxy-4-methylphenol was described as phenolic, smoke, plastic [15], candy, spice and smoked [29], wood, smoke and phenol [7]. One problem with those studies is that they did not indicate the concentration of these chemicals. The odor character of compounds changes at different concentrations [30,31]. Chambers et al. [32] and Ojeda et al. [33] used highly trained sensory panelists to describe the sensory characteristics of several phenolic compounds associated with smoked aroma characteristics, such as *p*-cresol, *m*-cresol, thymol, guaiacol, 4-ethylguaiacol, eugenol, isoeugenol, carvacrol and 2,6-dimethoxyphenol. No studies were found that used well-defined, smoky aroma attributes to describe chemical compounds at different concentrations by highly trained sensory panelists. Because chemical compounds exist at different concentrations in various food products, it is important to understand the aroma impact of those compounds as different concentrations.

The objective of this research was to: (1) describe the sensory characteristics of phenolic compounds that had been associated with smoked aroma chemical sources in the literature and (2) provide those descriptions at various concentration levels. Both objectives used a well-defined smoked aroma terms established by a highly trained sensory panel to describe the specific types of smoky aroma provided by those compounds.

## 2. Results and Discussion

Based on a list of attributes defined and referenced by Jaffe et al. [28] and used in this research (Table 1) panelists determined that a combination of various sensory attributes made up the smoked aromatic characteristics. The smoked aroma characteristics in most products was the result of a combination of smoky and one or more of the characterizing attributes such as woody, ashy, burnt, or cedar. 

The odor characteristics of all phenolic compounds illustrating the smoked characteristics are shown in Table 2. Most chemicals illustrated the smoked characteristics starting at low concentrations of 1 ppm and 10 ppm. There were two chemicals the panel described as having smoked characteristics starting at a higher concentration: 4-allyl-2,6-dimethoxyphenol was described as smoky at a concentration of 100 ppm, and 3,4-dimethylphenol at a concentration of 5000 ppm.

No smoky odor characteristics were found in 2,6-dimethylphenol. Although described with some smoked characteristics such as burnt or woody [29], 2,6-dimethylphenol was rather associated with non-smoky sensory attributes such as musty damp, sweet/perfume, pungent and chemical in our study. Because many studies examined effluent from gas chromatographs, differences in chemical purity or the ability to sniff for longer periods in this study may explain some differences from other literature [6,15,29]. It is possible that 2,6-dimethylphenol might produce smoked characteristics when combined with other chemicals, but combinations were not studied in this research.

In general, as the chemical concentration increased, the chemical odor characteristics became more intense. For example, the smoky attribute of 2,6-dimethoxyphenol increased from a score of 1 at 10 ppm to 5 at 100,000 ppm. However, some compounds did not have the highest score at the highest concentration. For instance, the highest score of smoky attribute of thymol is 3.5 at 1000 ppm, and no smoky attribute was found at 5000 ppm and higher.

More odor attributes were found in many chemicals as the concentration increased. For instance, 2,6-dimethoxyphenol was described as only smoky attribute at the concentration of 10 ppm and 100 ppm, while other odor attributes, such as woody or pungent were found when the concentration reached 1000 ppm and higher, and musty/dusty was found when the concentration reached 5000 ppm and higher.

The odor character of some compounds changed at different concentrations. For example, 2,4-dimethylphenol has only the smoky attribute at 1 ppm and 10 ppm, only the musty/dusty one at 100 ppm, but the smoky attribute appeared again when the concentration reached 1000 ppm and higher. The odor characteristics of 2-methoxy-4-vinylphenol at 10 ppm were unidentified, but other concentrations were described as having smoky characteristics.

Table 3 illustrates the odor profiles for the 10 phenol derivatives at the concentration best exhibiting the smoky characteristics. Note that some isomers have different characteristics. Among the four dimethyphenol isomers we evaluated in our study, no smoked characteristics were found in 2,6-dimethylphenol at any tested concentration, whereas 3,4-dimethylphenol at 1000 ppm and lower, and 2,5-dimethylphenol at 1 ppm did display such characteristics, and smoked characteristics were found in 2,4-dimethylphenol at all concentrations. Acrid and cedar attributes were found when *o*-cresol was 5000 ppm and higher, but were not founded in *m*-cresol and *p*-cresol. Only the smoky attribute was found when *p*-cresol was at 1 ppm and *o*-cresol at 10 ppm, but the petroleum-like attribute was found in *m*-cresol at all concentrations. 

Table 4 illustrates the odor profiles for the six guaiacol derivatives and two syringol derivatives at the concentration best exhibiting the smoked characteristics. Although Kostyra and Baryłko-Pikielna [10] suggested the possible contribution of guaiacol and 4-methylguaiacol to smoked flavor, but not of other guaiacol derivatives, we found by comparing parallel chemical and sensory characteristics of smoke flavorings that smoked characteristics were found in all six guaiacol derivatives. The score of smoky, woody, and cedar of isoeugenol were higher than that of eugenol, and a pungent attribute was found when isoeugenol was 5000 ppm and higher. A musty/earthy attribute was found when eugenol was at 1000 ppm and higher. The woody attribute was found in all six guaiacol derivatives and the scores for the woodiness of guaiacol and 2-methoxy-4-vinylphenol did not increase with the chemical concentration. There were only two syringol derivatives evaluated in this research, and both a smoky attribute and a woody attribute were found when the syringol derivatives were at 1000 ppm and higher, a pungent attribute was found when 2,6–dimethoxyphenol was at 1000 ppm and higher, and a cedar odor was found when 4-allyl-2, 6-dimethoxyphenol was at 5000 ppm and higher.

## 3. Materials and Methods

### 3.1. Panelists

Highly trained panelists from the Sensory Analysis Center at Kansas State University (Manhattan, KS, USA) took part in this research. All the panelists had completed 120 h of sensory descriptive training and each had more than 1000 h of testing experience, including smoked products. Each panelist had a broad background of experience in odor description and evaluation. Similar panelists have been usedrecently to test other sensory properties [34,35].

### 3.2. Chemicals

Nineteen phenolic compounds were used in this study. Phenolic compounds were the focus of the study because they have been regarded as the key compounds for smoky aroma. Although other compounds or, in fact, combinations of phenolic compounds could be studied, we focused only on the individual phenolic compounds in this initial study. All chemicals were selected because they have been referred to in previous literature to be potentially associated with smoked characteristic in various foods or because they had been found in the headspace of smoked products. Although phenol had been found in the headspace of smoked products frequently, phenol was not tested directly because phenol is toxic if inhaled and was been determined to be marine, metallic, chemical, and mushroom by gas chromatography−mass spectroscopy/olfactometry, commonly referred to as GC-MS/O [29]. The chemicals used were 11 phenol derivatives(*o*-cresol, *m*-cresol, *p*-cresol, thymol, 2,5-dimethylphenol, 3,4-dimethylphenol, 2,4-dimethylphenol, 2,6-dimethylphenol, 2,4,6-trimethylphenol, 3-ethyl-5-methylphenol, carvacrol), six guaiacol derivatives (guaiacol, 4-ethylguaiacol, 2-methoxy-4-methylphenol, 2-methoxy-4-vinylphenol, eugenol, isoeugenol), two syringol derivatives (2,6-dimethoxyphenol and 4-allyl-2,6-dimethoxyphenol). All these chemicals were available commercially (most chemicals were purchased from Fisher Scientific Co., Fair Lawn, NJ, USA, while carvacrol and 3-ethyl-5-methylphenol were purchased from Sigma-Aldrich Co., St. Louis, MO, USA) and were not considered toxic when used at low levels or in limited exposure as for sensory reference materials. 

### 3.3. Sample Preparation

All chemicals were diluted in propylene glycol (Sigma-Aldrich Co.) [30]. Seven concentrations were prepared by a serial dilution technique starting from a 100,000 ppm stock solution. The seven concentrations included 1, 10, 100, 1000, 5000, 10,000 and 100,000 ppm. To deliver the chemicals, a fragrance strip was dipped to a 1.25 cm depth into the specified chemical solution and placed into a 20-mL capped, coded glass tube. Chemical solutions and the fragrance strip preparation were completed approximately 24 h prior to testing.

### 3.4. Evaluation Procedure. All Chemicals Were Coded with 3-Digit Numbers and the Order in Which Chemicals Were Evaluated Was Randomized

A total of three sessions (4.5 h) were used to screen whether the 19 chemicals were found to have smoky characteristics that needed further evaluation. If both 1000 ppm and 100,000 ppm of a chemical was considered to have no smoky characteristics, the chemical did not need further evaluation. 

For chemicals that were considered to have smoked characteristics, an odor profile was determined for each concentration by each panelist using a lexicon [28] to describe smoked aroma characteristics. All dilutions (1–100,000 ppm) of a chemical were presented simultaneously and the panel evaluated them in the order from lowest to highest concentrations. One chemical was evaluated in each 1.5-h session.

A modified sensory profile method as described by Vara-Ubol et al. [30] and Chambers et al. [31] was used for the evaluation of the chemicals. Panelists evaluated each chemical concentration by taking quick sniffs from the fragrance testing strips. Each panelist examined samples individually, and then as a group, determining the odor profile of the chemical at the concentration. For profiling, a 15-point intensity scale was used, where 1 represents “just recognizable” and 15 represents “extremely intense”.

Five to 10 min elapsed between sniffing different concentrations to reduce carryover. Panelists stopped testing anytime carryover from one concentration to another could not be avoided. Fresh air and sniffs of a warm, wet towel were used to cleanse the nasal passages between samples.

## 4. Conclusions

The sensory panel described the smoked aromatic characteristics as being comprised of multiple sensory attributes. Most of phenolic compounds were described as smoky at low concentration, generally at 1 and 10 ppm, except 3,4-dimethylphenol at 5000 ppm. This shows the powerful impact of phenolic compounds on smokiness in foods. Smoky, woody and dusty/musty attributes were found in the odor characteristics of most phenolic compounds. Among the 19 phenolic compounds, only 2,6-dimethylphenol was not associated with any smoky characteristics. Further research needs to be conducted on other compounds as well as combinations of compounds.

## Figures and Tables

**Table 1:** List of sensory attributes imparting smoked aroma characteristics *.

Smoky	Ashy	Woody
Musty-Dusty	Musty/Earthy	Burnt
Acrid	Pungent	Petroleum-like
Creosote/Tar	Cedar	

* 11 attributes from Jaffe et al. [28] that are aroma active.

**Table 2:** Odor attributes of chemicals exhibiting smoky characteristics at different concentrations.

Chemicals	Odor Characteristics of Smoky Chemicals at Different Concentrations (ppm) *						
	1	10	100	1000	5000	10,000	100,000
**Phenol Derivatives**							
*m*-Cresol	smoky, petroleum-like	smoky, woody, musty/dusty, petroleum-like	smoky, musty/dusty, petroleum-like				
*o*-Cresol	smoky, woody, musty/dusty	smoky	smoky, ashy, woody, musty/dusty, acrid, pungent	smoky, musty/dusty, petroleum-like	smoky, musty/dusty, acrid, pungent, petroleum-like, cedar		
*p*-Cresol	smoky	smoky, musty/dusty	smoky, musty/dusty, petroleum-like	smoky, petroleum-like	smoky, musty/dusty, petroleum-like		smoky, musty/dusty, pungent, petroleum-like
2,5-Dimethylphenol	-	smoky, woody, musty/dusty		smoky, woody, musty/dusty, pungent		smoky, woody, musty/dusty, pungent, cedar	smoky, ashy, woody, musty/dusty, burnt, acrid, pungent, petroleum-like
2,4-Dimethylphenol	smoky		musty/dusty	Smoky, woody, musty/dusty	Smoky, woody, musty/dusty, pungent, cedar	Smoky, woody, musty/earthy, pungent, petroleum-like	Smoky, ashy, burnt, acrid, pungent
3,4-Dimethylphenol	-				smoky		smoky, woody, musty/dusty
3-Ethyl-5-methylphenol	smoky	smoky, woody, musty/dusty		smoky, woody			smoky, woody, acrid, pungent
Carvacrol	smoky	smoky, woody, musty/dusty, pungent, cedar			smoky, woody, musty/dusty, pungent, petroleum-like, cedar	smoky, woody, musty/dusty, pungent, cedar	smoky, woody, musty/dusty, pungent, petroleum-like, cedar
Thymol	smoky, woody		smoky, woody, musty/dusty, cedar	smoky, musty/dusty, pungent, petroleum-like	woody, musty/dusty, pungent, cedar	pungent	
2,4,6-Trimethylphenol	smoky, woody		smoky, musty/dusty			smoky, ashy, musty/earthy, burnt, acrid, pungent	
**Guaiacol Derivatives**							
Guaiacol	smoky, woody	smoky, woody, musty/dusty, petroleum-like		smoky, musty/dusty, pungent	smoky, musty/dusty, pungent, cedar		smoky, musty/earthy, pungent, cedar
4-Ethylguaiacol	smoky	smoky, woody		smoky, ashy, woody, burnt	smoky, woody	smoky, woody, pungent, cedar	smoky, ashy, woody, musty/dusty acrid, pungent, cedar
Eugenol	-	smoky	Smoky, musty/dusty	smoky, musty/dusty	smoky, woody, musty/earthy		
Isoeugenol	smoky, woody	smoky, woody, musty/dusty		smoky, woody, cedar	smoky, woody, pungent, cedar		
2-Methoxy-4-methylphenol	smoky, woody	smoky, woody, musty/dusty	smoky, woody, pungent		smoky, woody, musty/dusty, pungent		
2-Methoxy-4-vinylphenol	smoky	-	Smoky, woody	smoky			Smoky, woody, musty/dusty
**Syringol Derivatives**							
2,6-Dimethoxyphenol	-	smoky		smoky, woody, pungent	smoky, woody, musty/dusty, pungent		
4-Allyl-2,6-dimethoxyphenol	-		smoky, musty/dusty	smoky, woody, musty/dusty	smoky, woody, cedar		smoky, woody, pungent, petroleum-like, cedar

“-” indicates that the odor characteristics are unidentified. * The odor characteristics of each chemical are shown under specific concentrations.

**Table 3:** Odor profiles of phenol derivatives illustrating smoked characteristics with descriptions and intensities *.

*m*-Cresol at 100 ppm		*o*-Cresol at 1000 ppm	
smoky	2.5	smoky	3.0
musty/dusty	2.0	musty/dusty	2.5
petroleum-like	2.0	petroleum-like	2.0
chemical	2.5	chemical	2.5
*p*-Cresol at 100 ppm		2,4-Dimethylphenol at 1000 ppm	
smoky	1.5	smoky	2.5
musty/dusty	1.5	woody	2.0
petroleum-like	1.5	musty/dusty	2.0
2,5-Dimethylphenol at 10 ppm		3-Ethyl-5-methylphenol at 10 ppm	
smoky	2.0	smoky	2.0
woody	1.5	woody	1.5
musty/dusty	2.0	musty/dusty	1.5
Thymol at 100 ppm		Carvacrol at 10 ppm	
smoky	2.0	smoky	2.5
woody	2.0	woody	2.5
musty/dusty	1.5	musty/dusty	2.0
cedar	2.5	pungent	2.0
		cedar	2.5
2,4,6-Trimethylphenol at 10 ppm		3,4-Dimethylphenol at 5000 ppm	
smoky	2.0	smoky	2.0
woody	1.5		

* Intensity is based on a 15-point scale where 1 is “just recognizable” and 15 is “extremely intense”.

**Table 4:** Odor profiles of guaiacol derivatives and syringol derivatives illustrating smoked characteristics with descriptions and intensities *.

Guaiacol Derivatives			
Guaiacol at 100 ppm		2-Methoxy-4-methylphenol at 100 ppm	
smoky	3.5	smoky	3.5
woody	3.0	woody	2.0
musty/dusty	2.0	pungent	2.0
petroleum-like	1.5	sweet/floral	2.5
Eugenol at 5000 ppm		Isoeugenol at 5000 ppm	
Smoky	1.5	smoky	3.5
woody	2.0	woody	3.5
musty/earthy	2.5	pungent	2.0
pungent	2.0	cedar	3.5
cedar	2.0		
2-Methoxy-4-vinylphenol at 100 ppm		4-Ethylguaiacol at 100 ppm	
smoky	2.0	smoky	3.0
woody	1.5	woody	2.0
Syringol Derivatives			
2,6–Dimethoxyphenol at 1000 ppm		4-Allyl-2,6-dimethoxyphenol at 10 ppm	
smoky	3.5	smoky	2.0
woody	2.0	woody	1.5
pungent	1.5	musty/dusty	1.5

* Intensity is based on a 15-point scale where 1 is “just recognizable” and 15 is “extremely intense”.
